# Supplementary material for: Astroglial Hmgb1 regulates postnatal astrocyte morphogenesis and cerebrovascular maturation
Source: Nat Commun. 2023 Aug 16;14:4965. doi: 10.1038/s41467-023-40682-3 (PMC10432480; doi:10.1038/s41467-023-40682-3)
Supplement: Supplementary file 1 — Supplementary Information [file 41467_2023_40682_MOESM1_ESM.pdf]

# Supplementary Information

# Astroglial *Hmgb1* regulates postnatal astrocyte morphogenesis and cerebrovascular maturation

Moises Freitas-Andrade[1], Cesar H. Comin[2], Peter Van Dyken[3], Julie Ouellette[1,3], Joanna Raman-Nair[1,3], Nicole Blakeley[1,3], Qing Yan Liu[4,5], Sonia Leclerc[4], Youlian Pan[6], Ziying Liu[6], Micaël Carrier[7], Karan Thakur[1], Alexandre Savard[1], Gareth M. Rurak[8], Marie-Ève Tremblay[7], Natalina Salmaso[8], Luciano Da F. Costa[9], Gianfilippo Coppola[10], and Baptiste Lacoste[1,3,11]

[1] Neuroscience Program, The Ottawa Hospital Research Institute, Ottawa, ON, Canada
[2] Federal University of São Carlos, Department of Computer Science, São Carlos, Brazil
[3] Cellular & Molecular Medicine, University of Ottawa, Ottawa, ON, Canada
[4] National Research Council of Canada, Human Health and Therapeutics, Ottawa, Ontario, Canada
[5] Department of Biochemistry Microbiology and Immunology, Faculty of Medicine, University of Ottawa, Ottawa, Ontario, Canada
[6] Digital Technologies, National Research Council of Canada, Ottawa, Ontario, Canada
[7] Division of Medical Sciences, University of Victoria, BC, Canada
[8] Department of Neuroscience, Carleton University, Ottawa, ON, Canada
[9] University of São Paulo, São Carlos Institute of Physics, FCM-USP, São Paulo, Brazil
[10] Yale School of Medicine, Dept. of Pathology, New Haven, CT, USA
[11] University of Ottawa Brain and Mind Research Institute, Ottawa, ON, Canada

## This file contains:

- Supplementary figures
- Supplementary figure legends

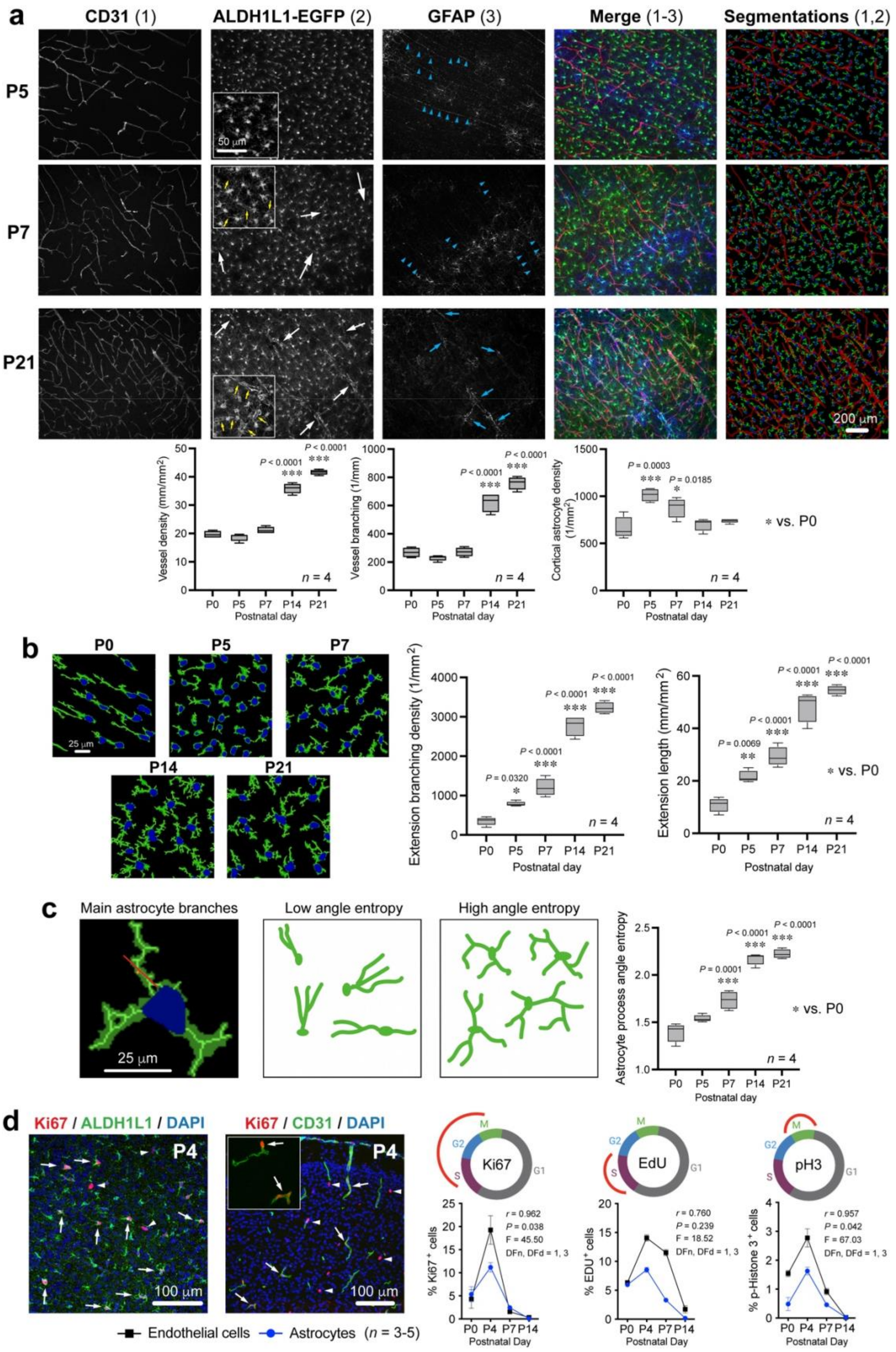
a
CD31 (1)
ALDH1L1-EGFP (2)
GFAP (3)
Merge (1-3)
Segmentations (1,2)
P5
P7
P21
50 μm
200 μm
Vessel density (mm/mm²)
Vessel branching (1/mm)
Cortical astrocyte density (1/mm²)
Postnatal day
P0 P5 P7 P14 P21
P < 0.0001
P = 0.0003
P = 0.0185
n = 4
* vs. P0
b
P0 P5 P7 P14 P21
25 μm
Extension branching density (1/mm²)
Extension length (mm/mm²)
P = 0.0320
P = 0.0069
P < 0.0001
n = 4
* vs. P0
c
Main astrocyte branches
Low angle entropy
High angle entropy
25 μm
Astrocyte process angle entropy
P = 0.0001
P < 0.0001
n = 4
* vs. P0
d
Ki67 / ALDH1L1 / DAPI
Ki67 / CD31 / DAPI
P4
100 μm
Ki67
EdU
pH3
G1 G2 M S
% Ki67⁺ cells
% EDU⁺ cells
% p-Histone 3⁺ cells
r = 0.962
P = 0.038
F = 45.50
DFn, DFd = 1, 3
r = 0.760
P = 0.239
F = 18.52
DFn, DFd = 1, 3
r = 0.957
P = 0.042
F = 67.03
DFn, DFd = 1, 3
Postnatal Day
P0 P4 P7 P14
Endothelial cells
Astrocytes (n = 3-5)

**Supplementary Fig. 1. The first two postnatal weeks are critical for astrocyte maturation.**

(**a**) Representative images of somatosensory cortical brain sections from *Aldh1l1-eGFP (BAC)* male mice at P5, P7 and P21. As seen in the merged image, sections were immunostained for the endothelial marker CD31 (red), and for astrocyte markers EGFP (green) and GFAP (blue). Blue arrowheads indicate radial glial processes. Segmentations (*far left*) represent reconstructed astrocytes and blood vessels that were quantified by automated analyses (see methods section for details) to evaluate vessel density and branching, as well as astrocyte cell density. (**b**) Representative images of segmented astrocytes during early postnatal brain development. Graphs show quantifications of astroglial branching density and extension length. Data are whisker boxes (min to max, center line indicating median). *$p < 0.05$, **$p < 0.01$, ***$p < 0.001$ (One-way ANOVA and Tukey's *post-hoc* test). (**c**) *Left*, Higher magnification showing a single astrocyte with a reference point in red by which the process angle entropy was calculated for main branches (see Methods section "Automated analysis of immunofluorescence images" for details). *Right*, Examples of low angle and high angle entropy is illustrated in panels on the right. Graph shows quantification of astrocyte process angle entropy across postnatal development. Data are whisker boxes (min to max, center line indicating median). *$p < 0.05$, **$p < 0.01$, ***$p < 0.001$ (One-way ANOVA and Tukey's *post-hoc* test). (**d**) Representative fluorescence micrographs of P4 cortical brain sections stained with proliferation marker Ki67 (red), ALDH1L1 (green, *left*), or CD31 (green, *right*) and the DNA stain DAPI (blue). The inset shows a close-up of proliferating endothelial cells. White arrows indicate proliferating astrocytes (*left*) or endothelial cells (*right*). White arrowheads point to non-astroglial or non-endothelial proliferating brain cells. Graphs show quantifications of proliferating astrocytes and endothelial cells during the first two postnatal weeks using various cell cycle markers. Correlation analyses was performed using Pearson correlation calculation ($p$ value based on two-tailed analysis). Data are mean ± SEM. All displayed microscopy images are representative of experiments repeated in 3-5 mice per group, with similar results. Source data are provided as a Source Data file.

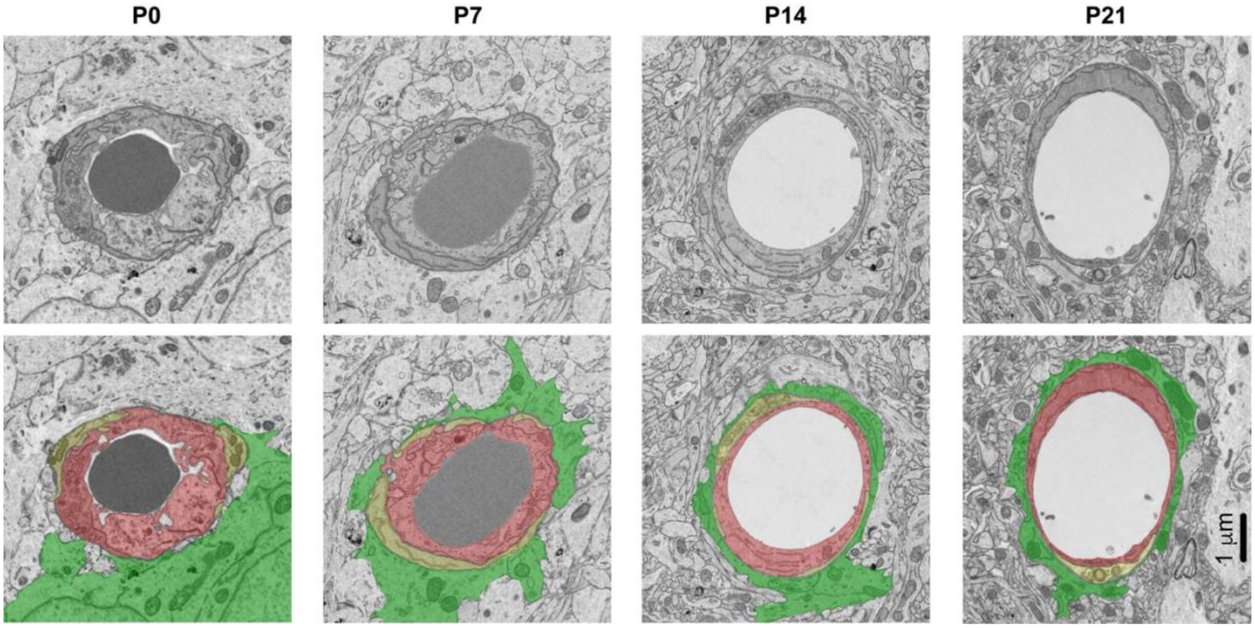


**Supplementary Fig. 2. Original electron micrographs (top) and unlabelled pseudo-coloured images (bottom).** All displayed microscopy images are representative of experiments repeated in 4 mice per group, with similar results.

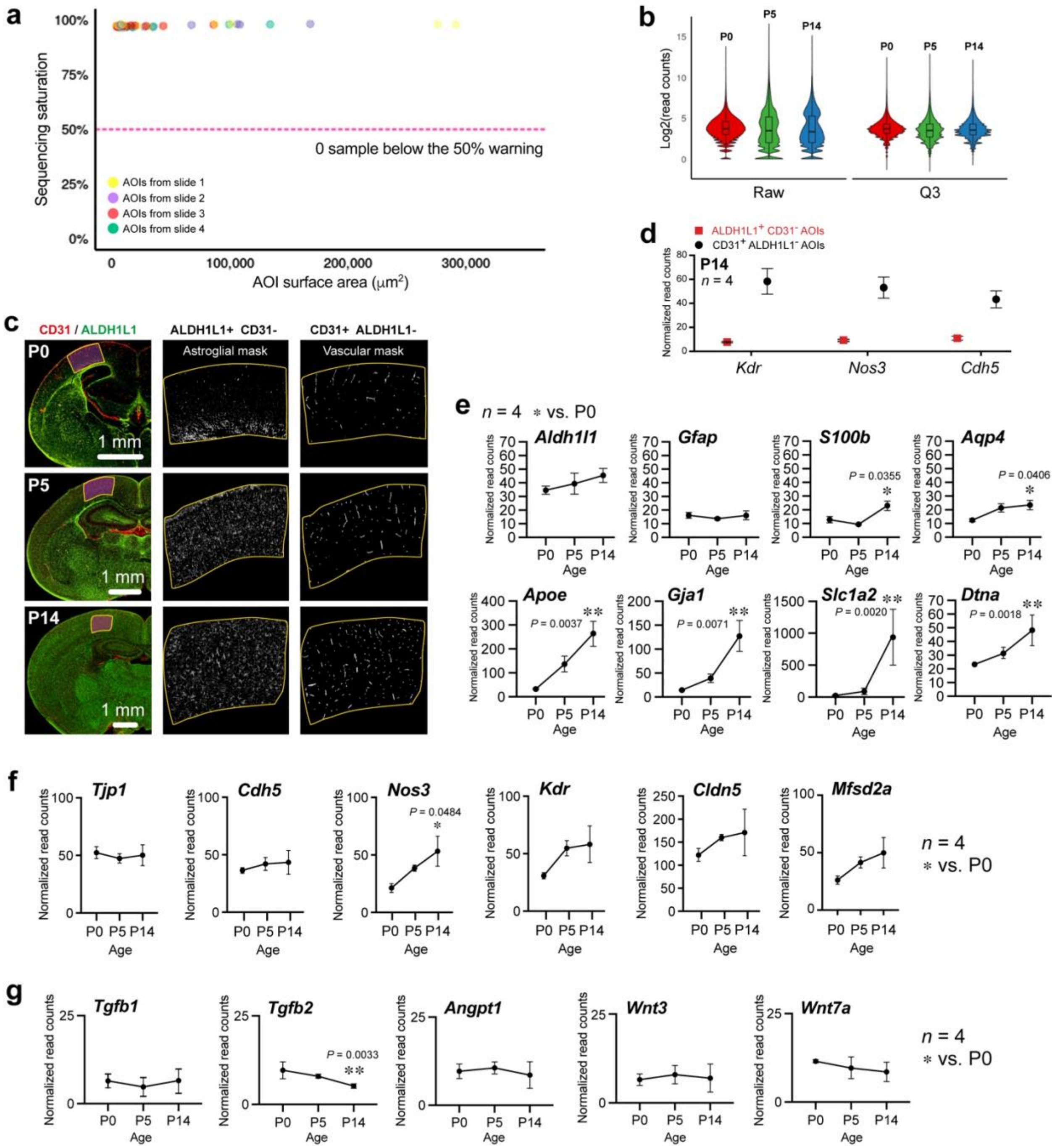

**Supplementary Fig. 3. Quality control for spatial transcriptomic data from astroglial and microvascular areas of interests during postnatal brain development.**

(**a**) Graph indicates sequencing saturation (sequencing depth) for each sample. Below 50%, the counts become less reliable. Our data set displays exceptionally high sequencing quality: no sample was found below the 50% warning. (**b**) Q3 normalization (Individual counts normalized against the 75th percentile of signal from their own area of interest, AOI) is used for the data set as it consistently outperforms other methods. In the graph, violin plots for each replicate (slide) illustrate changes in raw data after Q3 normalization (n=4 biologically independent animals per age). Each box plot displays the range between the first quartile and the third quartile of transcripts

within their respective groups. The center line indicates median and whiskers extend from the minimum to the maximum values found in each quartile. (**c**) *Left*, Representative tiled fluorescence micrograph of a brain section immunostained with ALDH1L1 (green) and CD31 (red) to select AOIs in the primary somatosensory cortex (S1) for multiplex spatial RNA sequencing at P0, P5 and P14 mice. Representative AOIs with astrocyte mask (*middle*) or microvascular mask (*right*) extracted for whole transcriptome profiling. (**d**) Graph indicates enrichment of vascular-specific genes in microvascular ($CD31^{+}ALDH1L1^{-}$) AOIs compared to astroglial ($ALDH1L1^{+}CD31^{-}$) AOIs at P14, supporting the validity of this method. (**e**) Graphs show expression (normalized read counts) of a set of astrocyte signature genes at P0, P5 and P14. (**f**) Graphs show expression (normalized read counts) of a set of endothelium signature genes at P0, P5 and P14. (**g**) Graphs show expression (normalized read counts) of a set of astroglial genes known as involved in angiogenesis. For **e-g**, n=4 biologically independent animals per age. All data in D-G are mean ± SEM. *$p < 0.05$, **$p < 0.01$, ***$p < 0.001$ (One-way ANOVA and Tukey's *post-hoc* test). Comparisons were made with respect to P0 values. All displayed microscopy images are representative of experiments repeated in 4 mice per group, with similar results. Source data are provided as a Source Data file. Raw sequencing data were deposited in GEO.

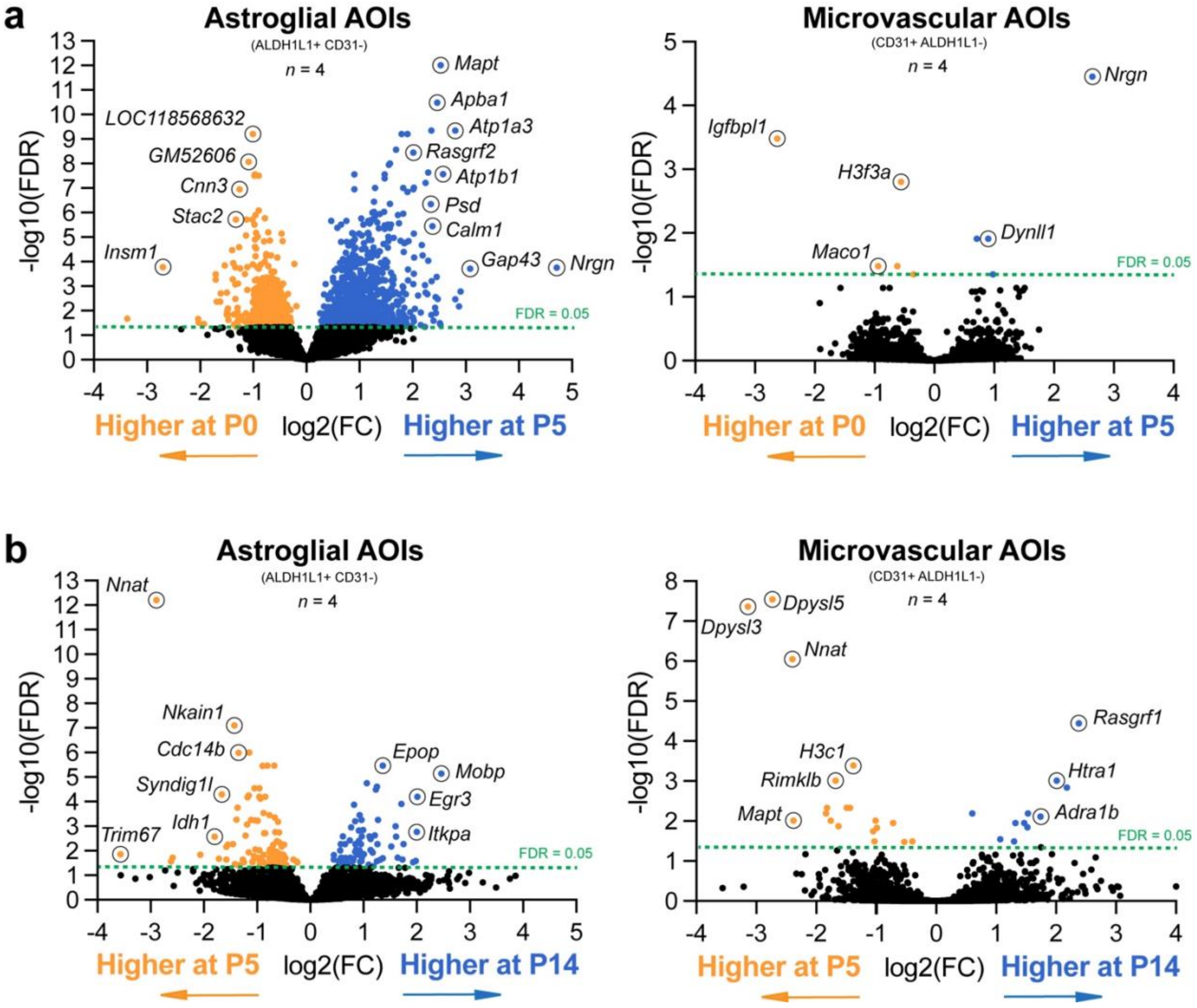


**Supplementary Fig. 4. *In situ* transcriptomic analysis of astrocytes and microvessels during the first two weeks of postnatal brain development.**

Volcano plots (False discovery rate, FDR, versus fold change, FC) displaying differentially-expressed genes in astrocyte-enriched and microvessel-enriched areas of interest (AOIs) between (**a**) P0 and P5, and between (**b**) P5 and P14. Raw sequencing data were deposited in GEO.

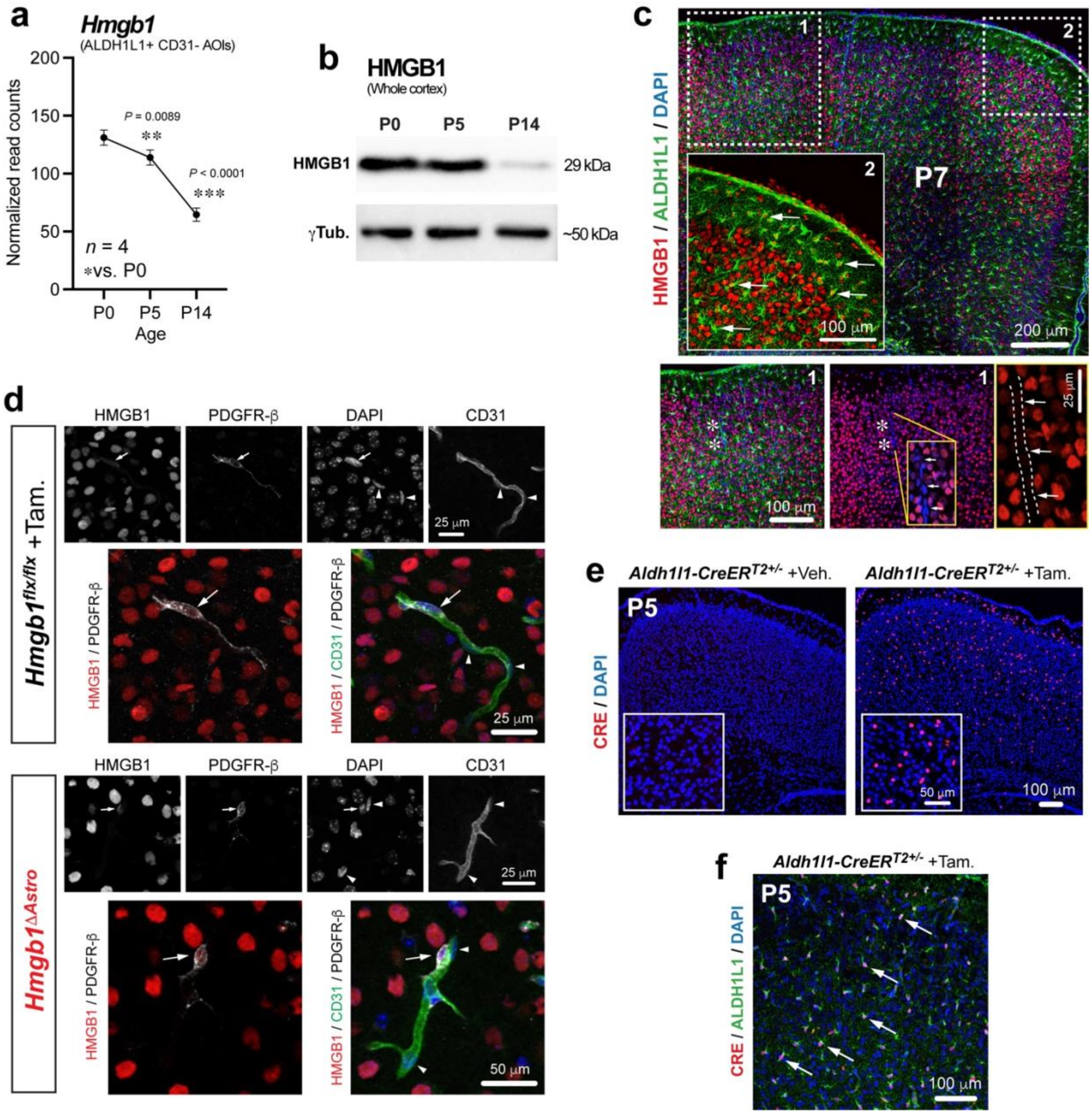


**Supplementary Fig. 5. *Hmgb1* expression in the cerebral cortex during postnatal development.**

(**a**) Graph highlights *Hmgb1* expression (normalized read counts) measured in astrocyte-enriched AOIs at P0, P5 and P14. Data are mean ± SEM. $^{**}p < 0.01$, $^{***}p < 0.001$ (One-way ANOVA and Tukey's *post-hoc* test). Comparisons were made with respect to P0 values. (**b**) Representative immunoblot of HMGB1 (upper blot) and loading control γ-tubulin (lower blot) from mouse cerebral cortex isolated at P0, P5 and P14. (**c**) Representative tiled fluorescence micrograph showing HMGB1 (red), ALDH1L1 (green) and DNA stain DAPI (blue) on brain sections from P7 mice. Box 1 outlines magnified areas (*bottom*) showing lack of HMGB1 in a microvessel (indicated by white asterisks and delineated by dotted lines). White arrows indicate endothelial nuclei. White arrows in box 2 point at astrocytes ($HMGB1^{+}$). (**d**) Representative fluorescence

micrographs of P14 cerebral cortex sections from in control (*top panels*) and *Hmgb1*$^{\Delta Astro}$ (*bottom panels*) mice immunostained for HMGB1 (red), CD31 (green) and pericyte marker PDGFR-b (grey), also stained with DNA marker DAPI (blue). White arrows point at pericytes. White arrowheads point at endothelial cell nuclei. (**e**) Representative fluorescence micrographs of cerebral cortex sections from vehicle-treated *Aldh1l1-CreER*$^{T2+/-}$ mice or Tamoxifen-treated *Aldh1l1-CreER*$^{T2+/-}$ mice, stained using DNA stain DAPI (blue) and a CRE antibody (red) at P5. Insets displayed higher magnifications. (**f**) Representative fluorescence micrographs of cerebral cortex sections from Tamoxifen-treated *Aldh1l1-CreER*$^{T2+/-}$ mice immunostained for CRE (red), ALDH1L1 (green), and stained with DNA marker DAPI (blue) at P5. White arrows indicate CRE recombinase immunoreactivity selectively in astroglial nuclei. All displayed microscopy images are representative of experiments repeated in 3-5 mice per group, with similar results. Source data are provided as a Source Data file.

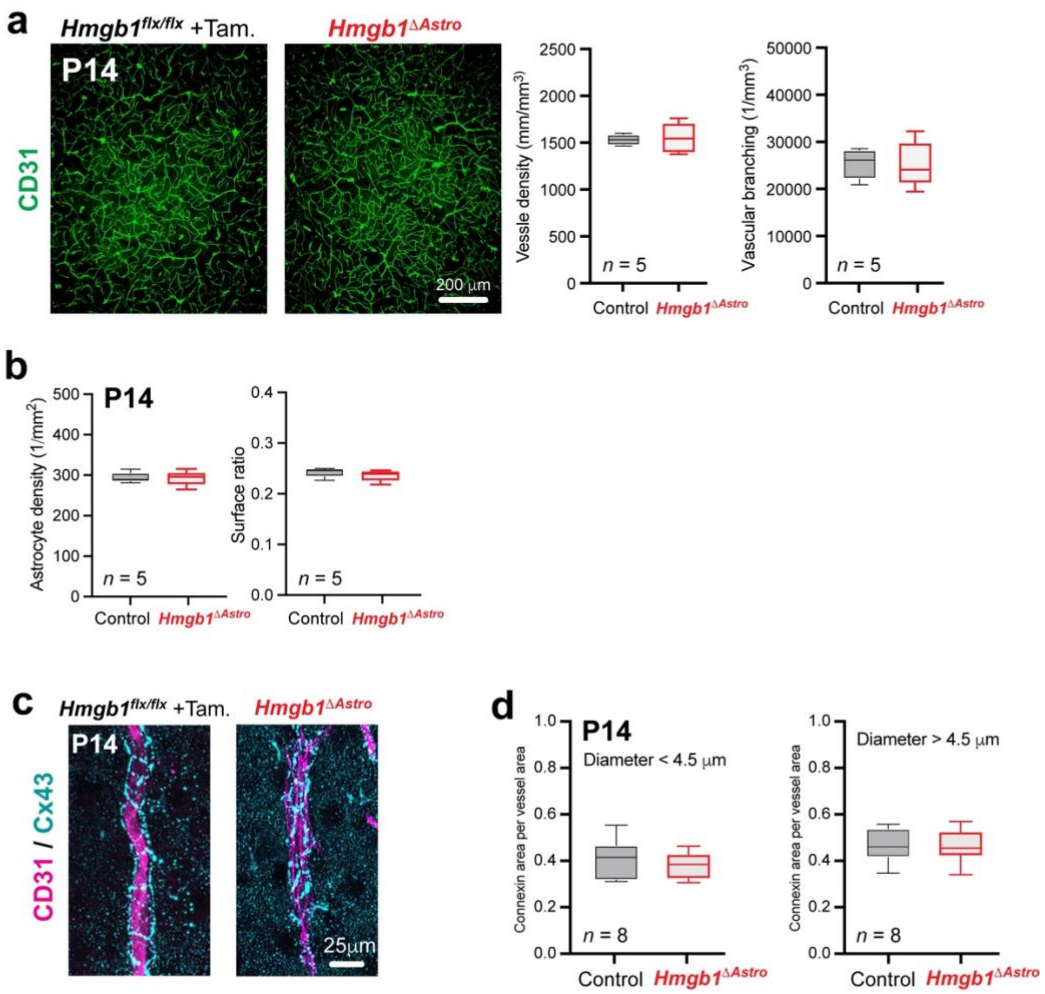


**Supplementary Fig. 6. Gliovascular parameters unaffected by loss of astroglial HMGB1.**

(**a**) Representative fluorescence micrographs showing CD31-positive microvessels (green) from control (*left*) or *Hmgb1*$^{\Delta Astro}$ (*right*) brain sections at P14. Quantifications of average vessel density (*left*) and branching (*right*) show no difference between groups. Data are whisker boxes (min to max, center line indicating median). (**b**) Quantification of average astrocyte density (*left*) and surface ratio (*right*) show no difference between genotypes. Data are whisker boxes (min to max, center line indicating median). (**c**) Representative fluorescence micrographs showing Cx43 immunoreactivity (cyan) on CD31-positive blood vessels (magenta) in cortical sections from control (*left*) or *Hmgb1*$^{\Delta Astro}$ (*right*) at P14. (**d**) Quantification of average Cx43-postivie area per vessel area, for microvessels of diameter < 4.5 µm (*left*) or > 4.5 µm (*right*) show no difference between genotypes. Data are whisker boxes (min to max, center line indicating median). All displayed microscopy images are representative of experiments repeated in 5 mice per group, with similar results. Source data are provided as a Source Data file.

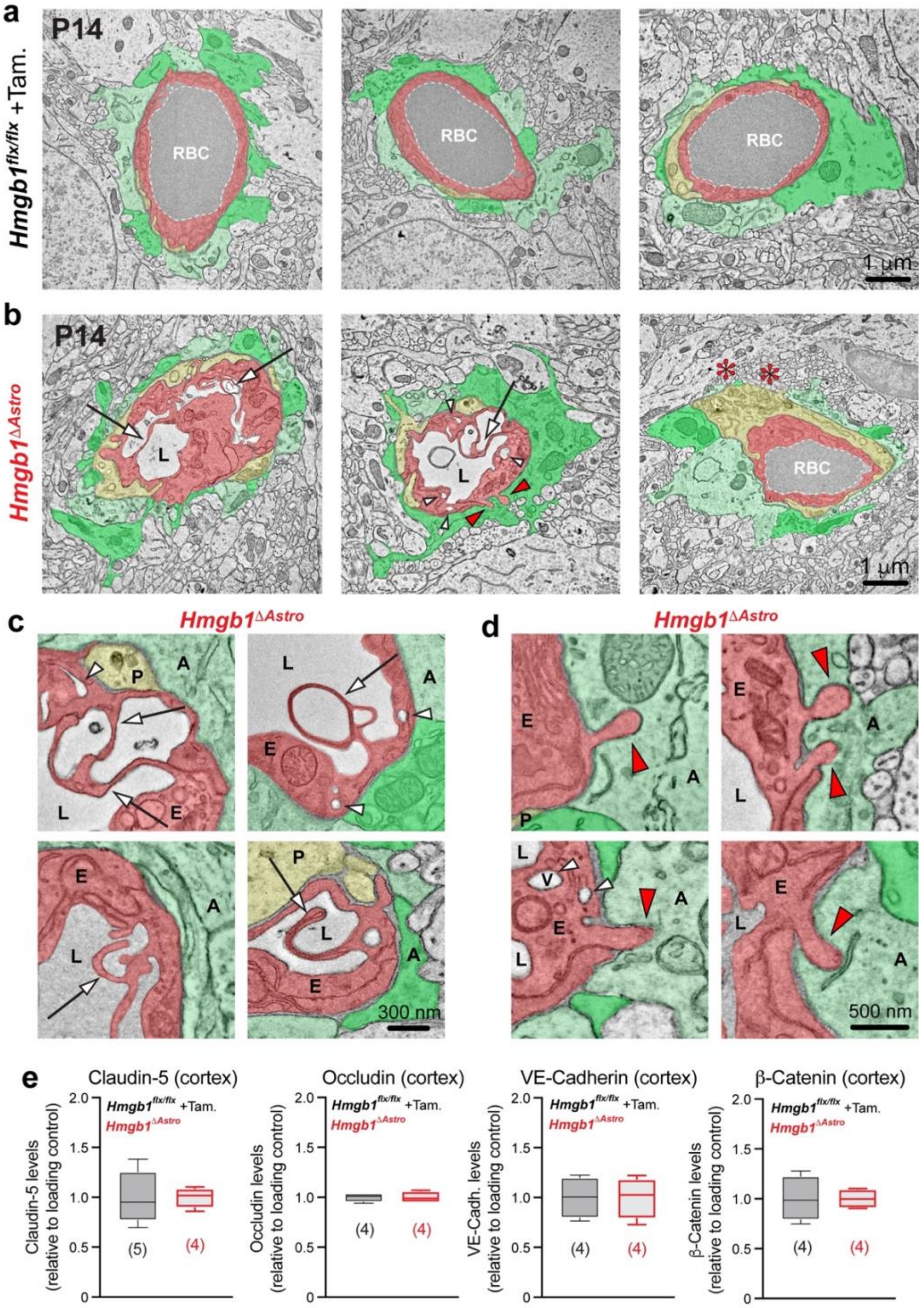


**Supplementary Fig. 7. HMGB1 ablation in newborn astrocytes affects gliovascular ultrastructure while the blood-brain barrier is maintained.**

(**a,b**) Representative low magnification electron micrographs showing ultrastructural features of astroglial endfeet (pseudocoloured in two shades of green for better separation) and endothelial cells (pseudocoloured in light red) from control (**a**) and in *Hmgb1*$^{\Delta Astro}$ (**b**) mice. Pericyte processes were pseudocoloured in light yellow. White arrows indicate macropynocytotic extensions; white

arrowheads indicate macropinocytotic vacuoles; red asterisks indicate regions of microvessel lacking endfoot coverage; red arrowheads point at endothelial protrusions extending into astroglial endfeet. (**c**) Representative higher magnification electron micrographs illustrating ultrastrucutral morphology of endothelial macropynocytotic extensions, indicated by white arrows, in *Hmgb1*$^{\Delta Astro}$ mice. White arrowheads indicate macropinocytotic vacuoles (V). (**d**) Higher magnification electron micrographs illustrating endothelial protrusions extending into astroglial endfeet, indicated by red arrowheads, in *Hmgb1*$^{\Delta Astro}$ mice. All electron micrographs were acquired in P14 animals. A, astrocyte; E, endothelium; L, lumen; P, pericyte. (**e**) Quantification of the levels of endothelial junction proteins (immunoblot signal normalized to the loading control and relative to control values, see Fig. 4d) show no difference between genotypes. Data are whisker boxes (min to max, center line indicating median). All displayed microscopy images are representative of experiments repeated in 4 mice per group, with similar results. Source data are provided as a Source Data file.

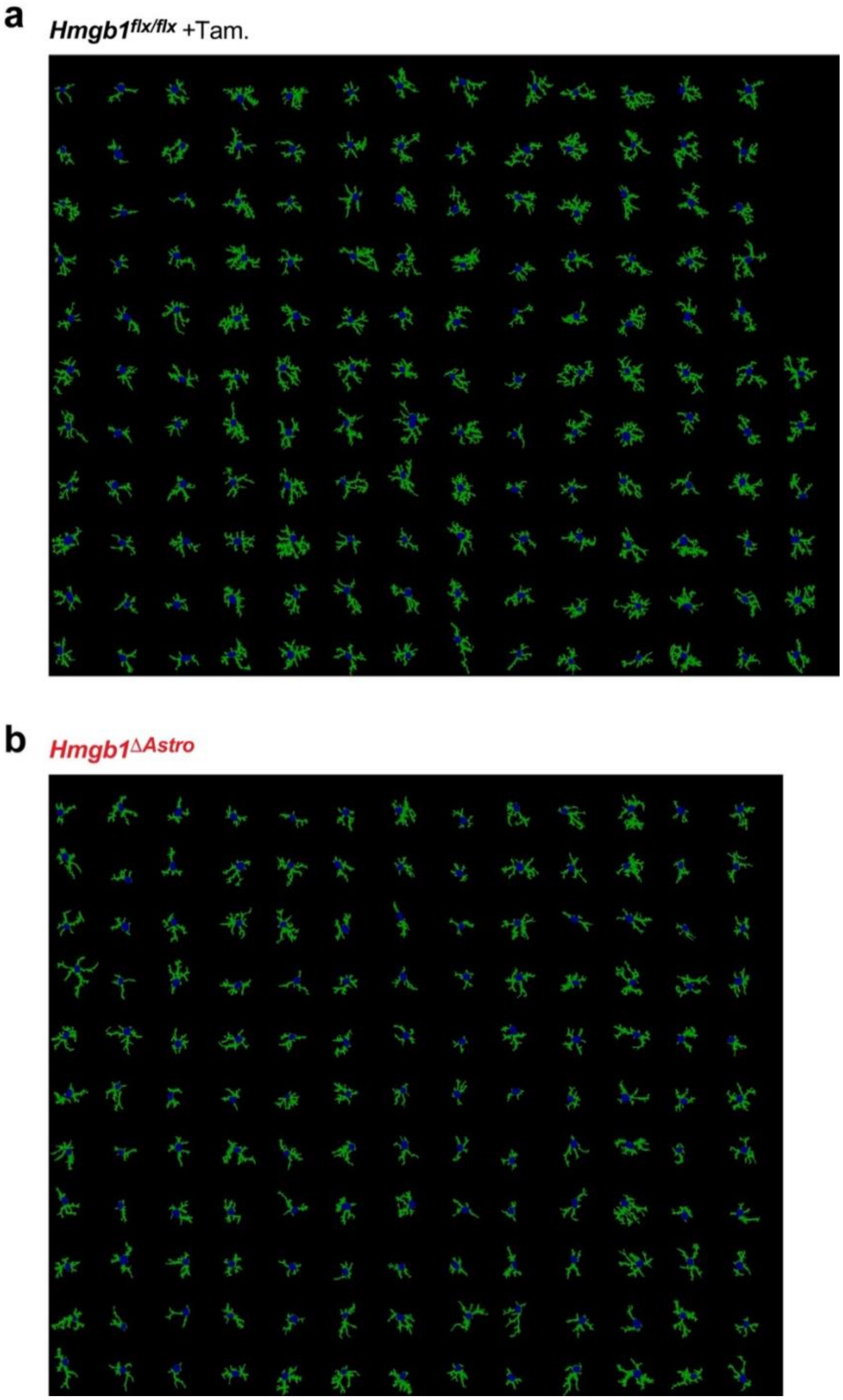


**Supplementary Fig. 8. Segmentation of individual astrocytes extracted from immunofluorescence images for in vivo morphological analysis.**

(**a**) All astrocytes segmented for the control group (n=149, from 4 animals). (**b**) All astrocytes segmented for the mutant group (n=143, from 4 animals).

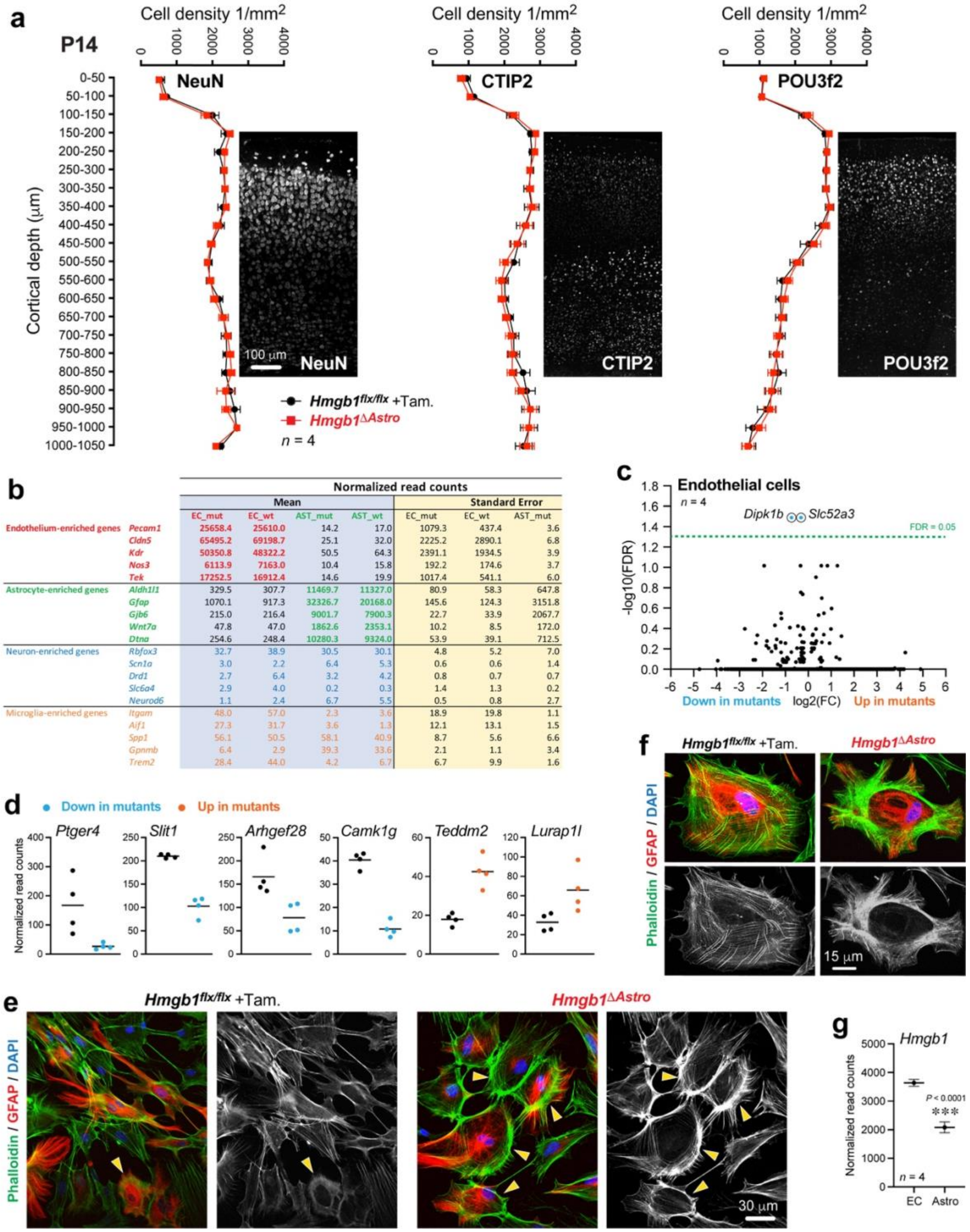


b

| | | Normalized read counts | | | | | | |
|---|---|---|---|---|---|---|---|---|
| | | Mean | | | | Standard Error | | |
| | | EC_mut | EC_wt | AST_mut | AST_wt | EC_mut | EC_wt | AST_mut |
| Endothelium-enriched genes | *Pecam1* | 25658.4 | 25610.0 | 14.2 | 17.0 | 1079.3 | 437.4 | 3.6 |
| | *Cldn5* | 65495.2 | 69198.7 | 25.1 | 32.0 | 2225.2 | 2890.1 | 6.8 |
| | *Kdr* | 50350.8 | 48322.2 | 50.5 | 64.3 | 2391.1 | 1934.5 | 3.9 |
| | *Nos3* | 6113.9 | 7163.0 | 10.4 | 15.8 | 192.2 | 174.6 | 3.7 |
| | *Tek* | 17252.5 | 16912.4 | 14.6 | 19.9 | 1017.4 | 541.1 | 6.0 |
| Astrocyte-enriched genes | *Aldh1l1* | 329.5 | 307.7 | 11469.7 | 11327.0 | 80.9 | 58.3 | 647.8 |
| | *Gfap* | 1070.1 | 917.3 | 32326.7 | 20168.0 | 145.6 | 124.3 | 3151.8 |
| | *Gjb6* | 215.0 | 216.4 | 9001.7 | 7900.3 | 22.7 | 33.9 | 2067.7 |
| | *Wnt7a* | 47.8 | 47.0 | 1862.6 | 2353.1 | 10.2 | 8.5 | 172.0 |
| | *Dtna* | 254.6 | 248.4 | 10280.3 | 9324.0 | 53.9 | 39.1 | 712.5 |
| Neuron-enriched genes | *Rbfox3* | 32.7 | 38.9 | 30.5 | 30.1 | 4.8 | 5.2 | 7.0 |
| | *Scn1a* | 3.0 | 2.2 | 6.4 | 5.3 | 0.6 | 0.6 | 1.4 |
| | *Drd1* | 2.7 | 6.4 | 3.2 | 4.2 | 0.8 | 0.7 | 0.7 |
| | *Slc6a4* | 2.9 | 4.0 | 0.2 | 0.3 | 1.4 | 1.3 | 0.2 |
| | *Neurod6* | 1.1 | 2.4 | 6.7 | 5.5 | 0.5 | 0.8 | 2.7 |
| Microglia-enriched genes | *Itgam* | 48.0 | 57.0 | 2.3 | 3.6 | 18.9 | 19.8 | 1.1 |
| | *Aif1* | 27.3 | 31.7 | 3.6 | 1.3 | 12.1 | 13.1 | 1.5 |
| | *Spp1* | 56.1 | 50.5 | 58.1 | 40.9 | 8.7 | 5.6 | 6.6 |
| | *Gpnmb* | 6.4 | 2.9 | 39.3 | 33.6 | 2.1 | 1.1 | 3.4 |
| | *Trem2* | 28.4 | 44.0 | 4.2 | 6.7 | 6.7 | 9.9 | 1.6 |

**Supplementary Fig. 9. Additional information about the impact of lack of astroglial HMGB1 on cortical maturation, gene expression and astrocyte morphology.**

(**a**) Quantification of neuronal densities with representative images in the somatosensory cortex following immunostaining for neuronal markers NeuN, CTIP2 or POU3f2 in *Hmgb1*$^{flox/flox}$ and *Hmgb1*$^{\Delta Astro}$ mice at P14. Data are mean ± SEM. (**b**) Quality control for the RNAseq experiment

shown as a table containing normalized read counts for selected genes. Data show high enrichment of endothelial genes in endothelial samples, high enrichment of astroglial genes in astroglial samples, and very low contamination by residual neuronal or microglial transcripts (n=4 animals per genotype). (**c**) Volcano plot (False discovery rate, FDR, versus Log2 fold change, FC) to visualize endothelial genes with differential expression at P14. Only two endothelial transcripts appeared as changed (FDR<0.05) among the 25,000+ detected genes. (d) Graphs reporting individual values and mean for astroglial DEGs of interest (FDR<0.05; FC>2; n=4 animals/samples per group). (**e**) Additional representative fluorescence micrographs showing phalloidin staining (F-actin, green) of GFAP-positive astrocytes (red) in primary cell cultures obtained from control (*left*) or $Hmgb1^{\Delta Astro}$ (*right*) mice at P14 (see Fig. 7f). Yellow arrowheads point at F-actin ring-like structures. (**f**) Higher magnifications of primary astrocyte cultures stained as in (**e**) to illustrate the remodeling of F-actin networks into a somatic ring in $Hmgb1^{\Delta Astro}$ astrocytes. Bottom images show desaturated phalloidin fluorescence only. (**g**) Graph reporting mean ± SEM for endothelial (EC) and astroglial (Astro) *Hmgb1* expression in control animals. ***$p$ < 0.001 (unpaired two-tailed Mann-Whitney's test). All displayed microscopy images are representative of experiments repeated in 3-4 mice per group, with similar results. Source data are provided as a Source Data file. Raw sequencing data were deposited in GEO.

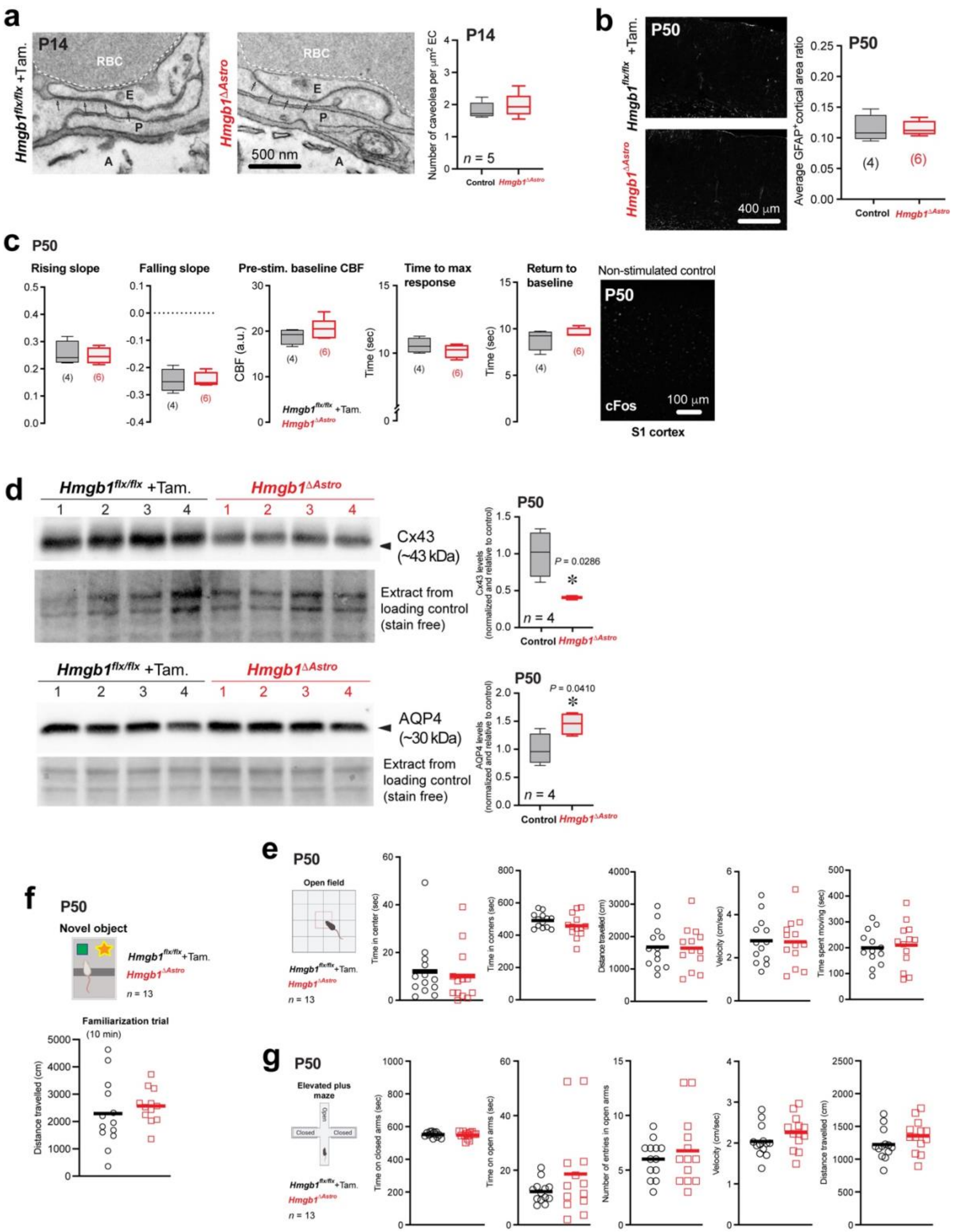


**Supplementary Fig. 10. Additional parameters of blood-brain barrier integrity, neurovascular function and behavior.**

(**a**) Representative electron micrographs illustrating ultrastructural features of endothelial tight junctions, indicated by small arrows, in control (*top*) and *Hmgb1*$^{\Delta Astro}$ (*bottom*) mice at P14.

E, endothelium; P, pericyte; A, astrocyte; RBC, red blood cell. Graph shows average total number of endothelial caveolae vesicles. (**b**) Immunostaining for Glial fibrillary acidic protein (GFAP) showed that cortical astrocytes in *Hmgb1*$^{\Delta Astro}$ are not reactive. Data are whisker boxes (min to max, center line indicating median). over the primary somatosensory S1 cortex in *Hmgb1*$^{flox/flox}$ and *Hmgb1*$^{\Delta Astro}$ mice at P50. Data are whisker boxes (min to max, center line indicating median). (**c**) Additional (unchanged) hemodynamic parameters of evoked CBF monitored over the primary somatosensory S1 cortex in *Hmgb1*$^{flox/flox}$ and *Hmgb1*$^{\Delta Astro}$ mice at P50. Data are whisker boxes (min to max, center line indicating median). (**d**) *Upper panels*, Cx43 immunoblot and loading control (*left*) from mouse cerebral cortex isolated at P50 in control (n=4, lanes 1-4) and age-matched *Hmgb1*$^{\Delta Astro}$ (n=4, lanes 5-8) mice. Graph (*right*) shows quantification of Cx43 immunoblot signal normalized to the loading control and relative to control group values. *Lower panels*, AQP4 immunoblot and loading control (*left*) from mouse cerebral cortex isolated at P50 in control (n=4, lanes 1-4) and age-matched *Hmgb1*$^{\Delta Astro}$ (n=4, lanes 5-8) mice. Graph (*right*) shows quantification of AQP4 immunoblot signal normalized to the loading control and relative to control group values. Data are whisker boxes (min to max, center line indicating median). $*p < 0.01$ (two-tailed Mann-Whitney's test). (**e**) Additional parameters from a 10-min open field task. No difference was detected between *Hmgb1*$^{\Delta Astro}$ and control littermates. data are mean with individual values. (**f**) Additional parameter (distance travelled, familiarization trial) from the novel object recognition task. No difference between *Hmgb1*$^{\Delta Astro}$ and control littermates was detected. data are mean with individual values. (**g**) Additional parameters from the elevated plus maze task. No difference was detected between *Hmgb1*$^{\Delta Astro}$ and control littermates. Data are mean with individual values. All displayed microscopy images are representative of experiments repeated in 4-6 mice per group, with similar results. Source data are provided as a Source Data file.

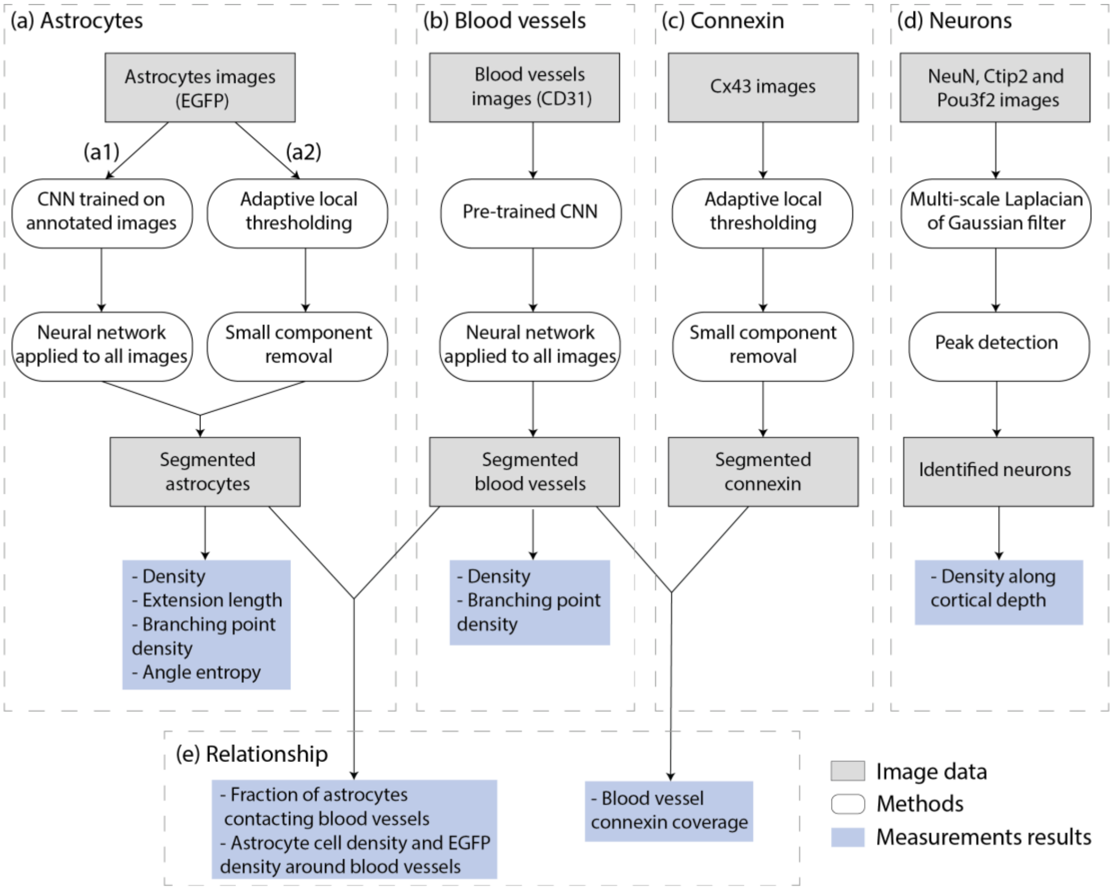


**Supplementary Fig. 11. Block diagram of the computerized methodology applied for the identification and analysis of:** (a) astrocytes, (b) blood vessels, (c) connexin, (d) neurons and (e) the relationship between astrocytes, blood vessels and connexin. Block (a) is subdivided into two main branches: (a1) astrocyte body identification and (a2) astrocyte processes identification.
