# Supplementary material for: Astroglial Hmgb1 regulates postnatal astrocyte morphogenesis and cerebrovascular maturation
Source: Nat Commun. 2023 Aug 16;14:4965. doi: 10.1038/s41467-023-40682-3 (PMC10432480; doi:10.1038/s41467-023-40682-3)
Supplement: Supplementary file 3 — Reporting Summary [file 41467_2023_40682_MOESM3_ESM.pdf]

nature portfolio

Corresponding author(s): Baptiste Lacoste

Last updated by author(s): Jun 26, 2023

Reporting Summary

Nature Portfolio wishes to improve the reproducibility of the work that we publish. This form provides structure for consistency and transparency in reporting. For further information on Nature Portfolio policies, see our Editorial Policies and the Editorial Policy Checklist.

Statistics

For all statistical analyses, confirm that the following items are present in the figure legend, table legend, main text, or Methods section.

n/a	Confirmed	
☐	☒	The exact sample size (*n*) for each experimental group/condition, given as a discrete number and unit of measurement
☐	☒	A statement on whether measurements were taken from distinct samples or whether the same sample was measured repeatedly
☐	☒	The statistical test(s) used AND whether they are one- or two-sided<br>*Only common tests should be described solely by name; describe more complex techniques in the Methods section.*
☐	☒	A description of all covariates tested
☒	☐	A description of any assumptions or corrections, such as tests of normality and adjustment for multiple comparisons
☐	☒	A full description of the statistical parameters including central tendency (e.g. means) or other basic estimates (e.g. regression coefficient) AND variation (e.g. standard deviation) or associated estimates of uncertainty (e.g. confidence intervals)
☐	☒	For null hypothesis testing, the test statistic (e.g. *F*, *t*, *r*) with confidence intervals, effect sizes, degrees of freedom and *P* value noted<br>*Give P values as exact values whenever suitable.*
☒	☐	For Bayesian analysis, information on the choice of priors and Markov chain Monte Carlo settings
☒	☐	For hierarchical and complex designs, identification of the appropriate level for tests and full reporting of outcomes
☐	☒	Estimates of effect sizes (e.g. Cohen's *d*, Pearson's *r*), indicating how they were calculated

Our web collection on statistics for biologists contains articles on many of the points above.

Software and code

Policy information about availability of computer code

Data collection	As we have described in the Method section, LabChart 8 software (ADInstruments, CO) was used for laser Doppler flowmetry and electrocorticography data collection. ZenPro 2.6 software (Carl-Zeiss-Strasse, Germany) was used with Zeiss Axio Imager M2 microscope equipped with a digital camera (Axiocam 506 mono) and the Zeiss ApoTome.2 module was used to collect immunohistochemistry and immunocytochemistry images. JEOL JEM-1400plus transmission electron microscope equipped with a Gatan digital camera was used to collect capillary imaging; Software used TEM center 1.7 for JEM-1400Flash and DigitalMicrograph 3.5 (Tokyo, Japan). Nanostring data collected by NanoString GeoMx® Digital Spatial Profiler 3.0.0.111 (GeoMx DSP) (Seattle, WA). Behaviour was recorded and analyzed using EthoVision 14 XT software program. Westen blot imaging collected using Image Lab software V3.5. RNA-Seq library sequencing was performed using FASTX-toolkit (http://hannonlab.cshl.edu/fastx_toolkit/).
Data analysis	ImageJ2 version: 2.3.0/1.53f (Bethesda, Maryland, USA) was used as well for analysis of immunofluorescent images. Nanostring data analyzed by Nanostring GeoMx DSP Data Analysis Suite (v2.4) [SEV-00090-05](Seattle, WA). Behaviour was recorded and analyzed using EthoVision software program 14 XT. LabChart 8 software (ADInstruments, CO) was used for laser Doppler flowmetry and electrocorticography data analysis. Computational morphometric analysis of 3D vascular images was completed using a Gaussian smoothing filter as well as a Palágyi-Kuba thinning algorithm (PMID:25155955) as described in the methods section. Automated analysis of immunofluorescence images performed with the use of Adam optimization algorithm and Palágyi-Kuba thinning algorithm. Analysis of neuronal density from 2D neuronal images was completed using the Laplacian of Gaussian (LoG) filter as described in the methods section. Graphpad Prism 9.0 & 9.3 software (GraphPad Software, CA). Western blot images analyzed using Image Lab software V3,5. RNA-Seq alignment of the reads, and normalization as well as identification of differentially expressed genes were performed using STAR (v2.7.9a) and DESeq2, respectively.

For manuscripts utilizing custom algorithms or software that are central to the research but not yet described in published literature, software must be made available to editors and reviewers. We strongly encourage code deposition in a community repository (e.g. GitHub). See the Nature Portfolio guidelines for submitting code & software for further information.

Data

Policy information about availability of data

All manuscripts must include a data availability statement. This statement should provide the following information, where applicable:

- Accession codes, unique identifiers, or web links for publicly available datasets
- A description of any restrictions on data availability
- For clinical datasets or third party data, please ensure that the statement adheres to our policy

All data supporting the findings of this study are available within the paper, its supplementary Information source data files, and deposited data and codes (see data/code availability statements).

Human research participants

Policy information about studies involving human research participants and Sex and Gender in Research.

Reporting on sex and gender	N/A
Population characteristics	N/A
Recruitment	N/A
Ethics oversight	N/A

Note that full information on the approval of the study protocol must also be provided in the manuscript.

Field-specific reporting

Please select the one below that is the best fit for your research. If you are not sure, read the appropriate sections before making your selection.

[x] Life sciences [ ] Behavioural & social sciences [ ] Ecological, evolutionary & environmental sciences

For a reference copy of the document with all sections, see nature.com/documents/nr-reporting-summary-flat.pdf

Life sciences study design

All studies must disclose on these points even when the disclosure is negative.

Sample size	No statistical method has been used to predetermine the sample size. All animal/sample/biological replicate numbers in this study are in line with well-accepted standards from the literature for each method. For instance, sample size was determine based on previous studies (PMID: 16319316, PMID: 24926076, PMID: 25155955, PMID: 24794428, PMID: 26051420, PMID: 27739237, PMID: 19749747, PMID: 27245461, PMID: 30528856, PMID: 29605426, PMID: 28009476, PMID: 25663600, PMID: 24429507, PMID: 33820803, PMID: 32661394). All in vitro, in vivo, and ex vivo experiments were designed according to these standards. Sample sizes chosen based on previous experiments/published work were sufficient to perform relevant analyses. Anatomical analysis for each genotype was perform from multiple tissue sections from a minimum of 4 mice (4-8 mice). Physiological measures were performed on at least 4 mice per group (4 to 5). Mouse behavior data for each genotype were sampled from a large number of mice (13 mice) in order to obtain sufficient statistical power to analyze differences between sexes (male and female mice were included in this study).
Data exclusions	No samples were excluded.
Replication	All data presented in this work were obtained from at least four experimental replicates (e.g., multiple animal cohorts from different litters; at least four experimental repeats for each assay, and biological replicates, etc.). All attempts of replication were successful.
Randomization	Groups were reassembled upon completion of data analysis according to genotype, age, sex. Randomization of individual samples/animals was performed by numbering.
Blinding	All experiments were performed blind.

Reporting for specific materials, systems and methods

We require information from authors about some types of materials, experimental systems and methods used in many studies. Here, indicate whether each material, system or method listed is relevant to your study. If you are not sure if a list item applies to your research, read the appropriate section before selecting a response.

Materials & experimental systems

n/a	Involved in the study	
☐	☒	Antibodies
☒	☐	Eukaryotic cell lines
☒	☐	Palaeontology and archaeology
☐	☒	Animals and other organisms
☒	☐	Clinical data
☒	☐	Dual use research of concern

Methods

n/a	Involved in the study	
☒	☐	ChIP-seq
☒	☐	Flow cytometry
☒	☐	MRI-based neuroimaging

Antibodies

Antibodies used

Primary antibodies used in this study:
Rat anti-CD31 (1:200) (BD pharmingen, CA) (Cat# 553370) (Lot # 8043575)
Guinea Pig anti-GFAP (1:500) (Synaptic Systems, Germany) (Cat # 173004) (Lot # 2-21)
Goat anti-IBA1 (1:300) (Abcam, MA) (Cat # ab107159) (Lot # 267451-3)
Goat anti-GFP (1:500) (Abcam, MA) (Cat # ab5450) (Lot # GR3200490-1)
Rabbit anti-Cre (1:1000) (Millipore Sigma, MO) (Cat # 69050) (Lot #2947865)
Guinea Pig anti-NeuN (1:1500) (Millipore Sigma, MO) (Cat # ABN90) (Lot # 3223348)
Rat anti-Ctip2 (1:200) (Abcam, MA) (Cat # ab18465) (Lot # GR3427932-4)
Rabbit Pou3F2 (1:200) (Cell Signaling, ON) (Cat # 12137S) (Lot # D2C1L)
Mouse anti-γ-tubulin (1:3000) (Thermo Fisher Scientific, ON) (Cat # MA1-850) (Lot #WJ337785)
Rabbit anti-HMGB1 (IF: 1:1000; WB: 1:2000) (Abcam, MA) (Cat # ab18256) (Lot #GR3299518-1)
Rabbit anti-Cx43 (IF: 1:400; WB: 1:5000) (Millipore Sigma, MO) (Cat # 6219) (Lot #0000127609)
Rabbit anti-Ki-67 (1:250) (Invitrogen, OR) (Cat # MA5-14520) (Lot #TD2549144A)
Rabbit anti-Phospho-Histone H3 (Ser10) (1:300) (Cell Signaling, ON) (Cat # 3377S) (Lot #D2C8)
Rabbit anti-AlDh1L1 (1:400) (Thermo Fisher Scientific, ON) (Cat # 702573) (Lot # BB246689)
Goat anti-AlDh1L1 (1:100) (Thermo Fisher Scientific, ON) (Cat # 600-101-HB6) (Lot # 42263)
Rabbit anti-c-Fos (1:1000) (Abcam, MA) (Cat # ab190289) (Lot # GR348522-1)
Rabbit anti-Aquaporin-4 (1:200) (Alomone Labs) (Cat # AQP-004) (Lot # AQP004AN2325)
Mouse anti-Aquaporin-4 (1:500) (Synaptic Systems) (Cat # 429011) (Lot #Cl325B5A5)
Alexa Fluor™ 488 Phalloidin (1:500) (Invitrogen, OR) (Cat # A12379) (Lot # 1816955)
Rabbit anti-Claudin-5 (1:1000) (Invitrogen, OR) (Cat # 34-1600) (Lot # W 1334125)
Rabbit anti-Occludin (1:1000) (Abcam, MA) (Cat # ab216327) (Lot # GR3243495-32)
Rabbit anti-beta Catenin (1:1000) (Abcam, MA) (Cat # ab32572) (Lot # GR184212-83)
Rabbit anti-Ve Cadherin (1:1000) (Abcam, MA) (Cat # ab205336) (Lot # GR229866-18)

Secondary antibodies used in this study:
Donkey anti-Rat IgG (H+L) Highly Cross-Adsorbed Secondary Antibody, Alexa Fluor 568 (1:300) (Invitrogen, OR) (Cat # A-11077) (Lot #1917936)
Donkey anti-Rat IgG (H+L) Highly Cross-Adsorbed Secondary Antibody, Alexa Fluor 488 (1:300) (Invitrogen, OR) (Cat # A-21208) (Lot #1979698)
Goat anti-Guinea Pig IgG (H+L) Highly Cross-Adsorbed Secondary Antibody, Alexa Fluor 594 (1:300) (Invitrogen, OR) (Cat # A-11076) (Lot # 2079357)
Donkey anti-Goat IgG (H+L) Cross-Adsorbed Secondary Antibody, Alexa Fluor 488 (1:300) (Invitrogen, OR) (Cat # A-11055) (Lot # 1942238)
Donkey anti-Rabbit IgG (H+L) Highly Cross-Adsorbed Secondary Antibody, Alexa Fluor 647 (1:300) (Invitrogen, OR) (Cat # A-31573) (Lot # 1964354)
Donkey anti-Mouse IgG (H+L) Highly Cross-Adsorbed Secondary Antibody, Alexa Fluor 568 (1:300) (Invitrogen, OR) (Cat # A-10037) (Lot # 1696197)
Goat anti-Guinea Pig IgG (H+L) Highly Cross-Adsorbed Secondary Antibody, Alexa Fluor 488 (1:300) (Invitrogen, OR) (Cat # A-11073) (Lot # 2087691)
Donkey anti-Rabbit IgG (H+L) Highly Cross-Adsorbed Secondary Antibody, Alexa Fluor 568 (1:300) (Invitrogen, OR) (Cat # A-10042) (Lot # 1891789)
Donkey anti-Goat IgG (H+L) Cross-Adsorbed Secondary Antibody, Alexa Fluor 488 (1:300) (Invitrogen, OR) (Cat # A-11055) (Lot # 1942238)
Anti-Rabbit IgG (H+L), HRP Conjugate (1:10000) (Promega, WI) (Cat # W4018) (Lot # 0000435308)
Goat anti-mouse HRP Conjugated (1:10000) (Thermo Fisher Scientific, ON) (Cat # PA1-74421) (Lot # YB3835674)

Validation

All antibodies were validated by the manufacturer or used in published research articles.

Rat anti-CD31 (BD pharmingen, CA) (PMID: 7956830) (manufacturer website states that this antibody has been validated with mouse brain tumors by Immunohistochemistry; Immunogen 129/Sv mouse-derived endothelioma cell line tEnd.1)
anti-GFAP (Synaptic Systems, Germany) (PMID: 34975768) (manufacturer website states that this antibody has been validated by KO, Reacts with: human, rat, mouse, chicken, sheep; Validate for Western blot, Immunocytochemistry, Immunohistochemistry, Immunohistochemistry of formalin fixed, paraffin embedded (FFPE) tissue)
Goat anti-IBA1 (Abcam, MA) (PMID: 30770053) (manufacturer website states This antibody has been validated with rat liver and

spleen tissue as well as marmoset kidney sections by Immunohistochemistry).
Guinea Pig anti-NeuN (Millipore Sigma, MO) (PMID: 25155955) (manufacturer website states This antibody has been validated with mouse cerebellum and frontal cortex by Immunohistochemistry).
Goat anti-GFP (Abcam, MA) (PMID: 30647151) (manufacturer website states that this antibody has been validated with mouse hindbrain tissue by Immunohistochemistry)
Rabbit anti-Cre (Millipore Sigma, MO) (PMID: 16998587) (manufacturer website states specific, sensitive detection of Cre recombinase in cells and cell lysates)
Rabbit Pou3F2 (Cell Signaling, ON) (PMID: 34657147) (manufacturer website states monoclonal antibody is produced by immunizing animals with a synthetic peptide corresponding to residues near the amino terminus of human Brn2/POU3F2 protein)
Rat anti-Ctip2 (Abcam, MA) (PMID: 32051547) (manufacturer website states tested by Flow Cyt: Jurkat cells. ICC: Neonatal mouse hippocampal cultured neurons WB: Nuclear extract from Jurkat cells; Mouse brain tissue lysate.)
Mouse anti-γ-tubulin (Thermo Fisher Scientific, ON) (PMID: 30872361) (manufacturer website states this Antibody was verified by Knockdown to ensure that the antibody binds to the antigen stated.)
Rabbit anti-HMGB1 (Abcam, MA) (PMID: 33373332) (manufacturer website states KO validated; Abpromise guarantee for the following applications: Immunocytochemistry, Immunofluorescence, Western blot; Reacts with Mouse, Rat and Human)
Rabbit anti-Cx43 (Millipore Sigma, MO)(PMID: 30872361) (manufacturer website states Anti-Connexin 43 reacts specifically with connexin 43. By immunoblotting, the antibody detects a single band or 2-3 bands at 43 kDa region. Staining of connexin 43 band(s) by immunoblotting is specifically inhibited with the connexin 43 peptide. Reactivity has been observed with human, bovine, rat, mouse, hamster and chicken connexin 43.)
Rabbit anti-Ki-67 (Invitrogen, OR) (PMID: 34093025) (manufacturer website states This Antibody was verified by Cell treatment to ensure that the antibody binds to the antigen stated.)
Rabbit anti-Phospho-Histone H3 (Ser10) (Cell Signaling, ON) (PMID: 35127720) (manufacturer website states Phospho-Histone H3 (Ser10) (D2C8) XP® Rabbit mAb detects endogenous levels of histone H3 only when phosphorylated at Ser10; however, this antibody does not detect phosphorylated Ser10 when Lys9 is acetylated or methylated. This antibody does not cross-react with histone H3 phosphorylated at Ser28.)
Rabbit anti-AlDh1L1 (Thermo Fisher Scientific, ON) (manufacturer website states This Antibody was verified by Relative expression to ensure that the antibody binds to the antigen stated; Advanced verification badge.) Antibody testing data:Immunofluorescence analysis of ALDH1L1 was performed using 70% confluent log phase RT4 cells; Immunofluorescence analysis, HeLa cells were fixed and permeabilized for detection of endogenous ALDH1L1 using ALDH1L1 Recombinant Rabbit Monoclonal Antibody; Western Blot was performed using Anti-ALDH1L1 Antibody (19H14L20), ABfinity Rabbit Monoclonal (Product # 702573) and a 99 kDa band corresponding to ALDH1L1 was observed across the cell lines and tissues tested except HaCa T, SK-OV-3, KARPAS 299, Hep G2, Mouse Heart, Mouse Brain and Rat Brain; Antibody specificity was demonstrated by detection of differential basal expression of the target across the cell lines and tissues tested owing to their inherent genetic constitution. Relative expression of ALDH1L1 was observed in RT4 cell line, Mouse Kidney, Mouse Liver and Rat Liver in comparison to HaCa T, SK-OV-3, KARPAS 299, Hep G2, Mouse Heart, Mouse Brain and Rat Brain using Anti-ALDH1L1 Antibody.
Goat anti-AlDh1L1 (Thermo Fisher Scientific, ON) (manufacturer website states This Antibody was verified by Immunofluorescence, immunohistochemistry and by Western Blot) Antibody testing data: Immunofluorescence Microscopy of Goat anti-Aldh1l1 antibody. Cells: HepG2 cells; Immunofluorescence Microscopy of Goat anti-Aldh1l1 antibody. Tissue: NIH3T3; Immunohistochemistry of Goat Anti-Aldh1l1 antibody. Tissue: mouse Liver tissue; Immunohistochemistry of Goat Anti-Aldh1l1 antibody. Tissue: mouse Kidney tissue; Western Blot of Goat Anti-Aldh1l1 Antibody. Lane 1: Opal Pre-stained MW ladder (p/n MB-210-0500). Lane 2: NIH3T3 Lysate; This affinity purified antibody is directed against mouse Aldh1l1. A BLAST analysis was used to suggest cross-reactivity with the antigen based on 100% homology with the immunizing sequence to human and rat.
Rabbit anti-c-Fos (Abcam, MA) (manufacturer website states Abpromise guarantee for the following applications: Immunohistochemistry-Free Floating, Immunocytochemistry, Western blot, Immunohistochemistry-Formalin/PFA-fixed paraffin-embedded sections; Reacts with Mouse, Rat, Human)
Rabbit anti-Aquaporin-4 (Alomone Labs) (manufacturer website states, antibodies are validated for specificity with the immunizing peptide in western blot analysis; can be used in western blot, immunocytochemical, immunohistochemical and indirect flow cytometry applications. It recognizes the Aquaporin 4 channel from rat, mouse and human samples. Knockout validation of Anti-Aquaporin 4 (AQP4) (249-323) Antibody in mouse retina.)
Alexa Fluor™ 488 Phalloidin (PMID: 18322103) (manufacturer website states Phalloidin is a high-affinity filamentous actin (F-actin) probe conjugated to our bright, photostable, green-fluorescent Alexa Fluor™ 488 dye)
Rabbit anti-Claudin-5 (1:1000) (Invitrogen, OR) (manufacturer website states synthetic peptide derived from the C-terminal region of mouse Claudin-5; Species Reactivity Human, Mouse; Tested for the following application: Western blot, Immunocytochemistry, Immunofluorescence, ELISA)
Rabbit anti-Occludin (1:1000) (Abcam, MA) (manufacturer website states knockout validated; Reacts with: Mouse, Rat, Dog, Human; Suitable for: Western blot, Immunocytochemistry, Immunofluorescence, Immunohistochemistry, Flow Cyto.)
Rabbit anti-beta Catenin (1:1000) (Abcam, MA) (manufacturer website states knockout validated; Reacts with: Mouse, Rat, Human; Suitable for: Western blot, Immunocytochemistry, Immunofluorescence, Immunohistochemistry, Immunoprecipitation)
Rabbit anti-Ve Cadherin (1:1000) (Abcam, MA) (manufacturer website states knockout validated; Reacts with: Mouse; Suitable for: Western blot, Immunocytochemistry, Immunofluorescence, Immunohistochemistry, Immunoprecipitation)

Donkey anti-Rat IgG (H+L) Highly Cross-Adsorbed Secondary Antibody, Alexa Fluor 488 (Invitrogen, OR) (PMID: 28280459)
Goat anti-Guinea Pig IgG (H+L) Highly Cross-Adsorbed Secondary Antibody, Alexa Fluor 594 (Invitrogen, OR) (PMID: 27100625)
Donkey anti-Goat IgG (H+L) Cross-Adsorbed Secondary Antibody, Alexa Fluor 568 (Invitrogen, OR) (PMID: 28266911)
Donkey anti-Rabbit IgG (H+L) Highly Cross-Adsorbed Secondary Antibody, Alexa Fluor 647 (Invitrogen, OR) (PMID: 28018179)
Donkey anti-Mouse IgG (H+L) Highly Cross-Adsorbed Secondary Antibody, Alexa Fluor 568 (Invitrogen, OR) (PMID: 28220786)
Goat anti-Guinea Pig IgG (H+L) Highly Cross-Adsorbed Secondary Antibody, Alexa Fluor 488 (Invitrogen, OR) (PMID: 28066195)
Donkey anti-Rabbit IgG (H+L) Highly Cross-Adsorbed Secondary Antibody, Alexa Fluor 568 (Invitrogen, OR) (PMID: 28367981)
Donkey anti-Goat IgG (H+L) Cross-Adsorbed Secondary Antibody, Alexa Fluor 488 (Invitrogen, OR) (PMID: 28186168)
Anti-Rabbit IgG (H+L), HRP Conjugate (1:10000) (Promega, WI) (PMID: 22389506)
Goat anti-mouse HRP Conjugated (1:10000) (Thermo Fisher Scientific, ON) (PMID: 36424919)

Animals and other research organisms

Policy information about studies involving animals; ARRIVE guidelines recommended for reporting animal research, and Sex and Gender in Research

Laboratory animals	All mice were bred in house. Breeders were 6-8 weeks old. Experimental animal were newborn (P0), P4, P5 P7, P14 P21 or P50, males and females. Aldh1l1-eGFP (BAC) males (Jackson laboratory, Stock No. 026033; B6 background) were crossed with WT "Noncarrier" females (Jackson laboratory). Conditional knockout of Hmgb1 was achieved by breeding Aldh1L1-Cre/ERT2 BAC transgenic male animals 1 (Jackson laboratory, Stock No. 031008; C57BL/6N-congenic background) into a background of female mice carrying a loxP-flanked Hmgb1 gene (Hmgb1flx/flx) 2 (Jackson laboratory, Stock No: 031274; B6 background).
Wild animals	Study did not involve wild animals.
Reporting on sex	Males and females were used in the study. Sex specific effects were not tested.
Field-collected samples	Study did not involve samples collected from the field.
Ethics oversight	All animal procedures were approved by the University of Ottawa Animal Care Committee and were conducted in accordance to guidelines of the Canadian Council on Animal Care. Generally, mice are on a 12/12 light cycle (7AM On / 7PM Off). Mice undergoing behavioral testing are on an inverted light cycle. Animal temperature for rodent rooms is 21C-23C, with humidity of 40-60%.

Note that full information on the approval of the study protocol must also be provided in the manuscript.
